# Efficacy of Systemic Biologic Drugs in Pediatric Psoriasis: Evidence From Five Selected Randomized Clinical Trials

**DOI:** 10.3389/fphar.2022.847308

**Published:** 2022-04-05

**Authors:** Vito Di Lernia, Laura Macca, Lucia Peterle, Ylenia Ingrasciotta, Gianluca Trifirò, Claudio Guarneri

**Affiliations:** ^1^ Dermatology Unit, Arcispedale S. Maria Nuova, Azienda USL-IRCCS di Reggio Emilia, Reggio Emilia, Italy; ^2^ Department of Clinical and Experimental Medicine, Section of Dermatology, University of Messina, Messina, Italy; ^3^ Department of Biomedical and Dental Sciences and Morpho-functional Imaging, Section of Pharmacology, University of Messina, Messina, Italy; ^4^ Department of Diagnostics and Public Health, Section of Pharmacology, University of Verona, Verona, Italy; ^5^ Department of Biomedical and Dental Sciences and Morphofunctional Imaging, Section of Dermatology, University of Messina, Messina, Italy

**Keywords:** psoriasis, paediatric, childhood, treatment, therapy

## Abstract

**Background:** Psoriasis is a chronic, immune-mediated skin disease that may occur at any age. Prevalence in children ranges between 0.5 and 1.0% across Europe. Approximately 10–20% of paediatric psoriasis patients are moderate-to-severe in severity and may require the use of systemic therapy.

**Objective:** Recently, newer targeted, systemic therapies have been licensed for treatment of moderate-to-severe paediatric psoriasis. The objective of this study was to evaluate the short-term efficacy of available antipsoriatic systemic drugs in children with a narrative synthesis of key efficacy from randomized clinical trials.

**Methods:** A systematic review of literature was performed on Medline and embase databases and the Cochrane Central Register of Controlled Trials. Randomized clinical trials investigating the efficacy of treatments licensed by the US Food and Drug Administration and/or the European Medicines Agency for paediatric and adolescent psoriatic population were retrieved and analyzed. Data from this literature review was assessed in line with GRADE (grading of recommendations, assessment, development and evaluations). The short-term (12-16 weeks) clinical efficacy from baseline was evaluated according to the Psoriasis Area and Severity Index (PASI) 75 and 90 compared to baseline. Illustrative comparative risks, relative risk (RR) and the number needed to treat (NNT) for response on PASI 75 and PASI 90 were extracted.

**Results:** A total of five relevant studies were identified on two TNF-alpha blockers (etanercept and adalimumab), the IL12/23 inhibitor ustekinumab and two IL-17 inhibitors (ixekizumab, secukinumab). Comparators were placebo (3 studies), placebo and etanercept (1 study) methotrexate (1 study). All examined drugs resulted efficacious. The probability to achieve PASI 75 and PASI 90 was higher for the IL-12/23 and IL-17 inhibitors. Overall, the anti-IL17s and the anti-IL12/23 antibodies showed a more favourable NNT for PASI 75, whereas IL-17 inhibitors for PASI 90.

**Conclusion:** The approved biological therapies may be beneficial for the treatment of moderate to severe plaque psoriasis in children and adolescents. Since psoriasis is a chronic and often challenging condition with no definitive solution, systematic evaluations of long-term efficacy, drug survival and adverse effects may help careful, individualized, patient-centered clinical decision making.

## Introduction

Psoriasis is a chronic inflammatory cutaneous disorder affecting 2-4% of the world’s population (Parisi et al., 2013; Ingrasciotta et al., 2021). Onset of psoriasis in infancy and adolescence is relatively common. Prevalence among children ranges between 0.5 and 1% across Europe and the median in individuals younger than 18 years is about 0.7% (Augustin et al., 2010). Psoriasis can appear at any age and all age groups may be affected (Augustin et al., 2010; Tollefson et al., 2010). The prevalence rates of the disease in childhood and adolescence are expected to increase in an approximately linear manner from the age of 1 year to the age of 18 years. The median prevalence in individual younger than 18 years is about 0.7% (Augustin et al., 2010). Therefore, onset of psoriasis in infancy and adolescence is relatively common (Raychaudhuri and Gross, 2000).

Although psoriasis severity in children is mild in the majority of patients, moderate to severe psoriasis can and do occur in children as well. Disease severity assessment is similar to adults. The Psoriasis Area and Severity Index (PASI), and the Body Surface Area (BSA) are the currently available tools for measurement of psoriasis severity in childhood as well (Spuls et al., 2010; Cannavò et al., 2017; Lavaud and Mahé, 2019). Skin symptoms are associated with significant impact on quality of life (QOL) for the patients and their parents and carers (Kim and Fischer, 2021). The Children Dermatology Life Quality Index (CDLQI) is considered a validated tool for measuring the impact of psoriasis in children. The majority of cases of psoriasis is mild and easily managed with topical treatments. Children with extensive lesions require systemic treatment and/or phototherapy. Validated definition of “severe disease” include the presence of at least one of the following criteria: PASI >10, BSA >10, CDLQI >10, according to the so-called ‘‘rule of tens” (Finlay, 2005). In addition, the involvement of sensitive and visible skin areas with high impact on QOL, as face, hands and feet, nails, intertriginous areas must be taken into account when evaluating the clinical severity of the disease (Fortina et al., 2017; Gisondi et al., 2021).

Severe psoriasis requires continuous systemic treatment with an effective and safe long-term therapy in consideration of the long-lasting natural history of the disease (Marcianò et al., 2020; Talamonti et al., 2020). However, the treatment of severe psoriasis in children may be challenging. Despite the lack of evidence for the non-biologic therapeutic options for paediatric psoriasis, these therapies have been featured prominently in the armamentarium of clinicians and a number of case series have been published (van Geel et al., 2015; Di Lernia et al., 2016a; Di Lernia et al., 2016b; Charbit et al., 2016; Di Lernia et al., 2017). This management may be driven by the extrapolation of evidence in adult psoriasis, the perceived risk-benefit profile of these therapies and the lack of the availability of medicines with an appropriate label for paediatric use. Indeed, methotrexate, cyclosporin, acitretin have not been evaluated formally among children and adolescents and are not licensed for paediatric psoriasis. Two TNF-alpha blockers, namely etanercept and adalimumab, were approved by European Medicine Agency (EMA) for the treatment of severe psoriasis in children (>6 years for etanercept, > 4 years for adalimumab), while only one of them, etanercept, was approved by the United States (US) Food and Drug Administration (FDA) in children >4 years. Ustekinumab, an anti-IL12/23 antagonist, was firstly been approved by the EMA and FDA for treatment of moderate to severe plaque psoriasis for children ≥12 years old. After the evaluation of the safety, efficacy, and pharmacokinetics in an open-label, single-arm, study in younger patients, (Philipp et al., 2020), ustekinumab use was expanded by the EMA and FDA also in patients from 6 to 11 year-old of age (Cvenkel and Starbek Zorko, 2021). More recently two biologic drugs targeting the IL-17A pathway, secukinumab and ixekizumab, were approved by the EMA and FDA as new treatment options for the treatment of moderate to severe psoriasis in patients aged 6–17 years.

The aim of this systematic review is to summarise the evidence on the efficacy of systemic treatments for moderate-to-severe psoriasis in children and adolescent patients based on randomized controlled trials (RCTs). This revision of studies does not focus on safety issues. Invaluable data about will be generated by long term observations, post marketing reviews and registries, since information on really relevant data cannot be drawn by RCTs.

## Materials and Methods

We conducted a systematic literature review of RCTs for the evaluation of treatments for moderate-to-severe psoriasis in children and adolescents. This study was organized according to the preferred reporting items for systematic review and meta-analysis protocols (PRISMA-P) 2015 statement (Moher et al., 2015).

### Literature Search

A literature search was implemented to identify pertinent articles published in PubMed, embase, and the Cochrane Central Register of Controlled Trials. The main search terms were “psoriasis” and “paediatric”. The literature search was limited to English and French language and human studies. In addition, the references of these articles were also examined for additional related articles.

### Study Selection

The inclusion and exclusion criteria were defined before the search. Eligible trials were required to 1) be randomized clinical trials (RCT) on treatments for moderate-to-severe chronic plaque psoriasis; 2) have study participants younger than 18 years of age; 3) evaluate pharmacologic interventions limited to systemic drugs licensed by the EMA or US FDA; and 4) examine the efficacy and safety of antipsoriatic drugs for plaque psoriasis as measured by changes from baseline in the PASI and indicate the proportions of patients who achieved at least a 75% reduction in PASI by the end of the primary response period (short-term: 12–16 weeks from baseline). As comparators, we accepted any other type of management for psoriasis, including placebo or active surveillance.

We accepted studies published in English and French without date restrictions. Non-randomized trials were excluded. Studies were not included if they were only available as abstracts from conference proceedings or if published in a language other than English and French, or if they were long-term extensions or analysis of already selected studies. As this is a review of previously published results of clinical trial data, no institutional board review was required. This article is structured on previously published studies and does not include any new studies with human participants performed by any of the authors.

### Types of Outcome Measures

Investigator-assessed improvement: proportion of participants achieving PASI 75 and PASI 90.

### Selection of Studies

Two review authors (VD and CG) independently checked abstracted data using a predefined data extraction form for all included studies. The full text of potentially relevant studies for assessment was retrieved. Both review authors independently judged if, from reading the full text, each study met the predefined selection criteria.

### Data Extraction and Analysis

Two review authors (VD and CG) performed data extraction. The following information was extracted from each study: author, year of publication, study type, study time frame, type of population (minimal age); sample size; the specific active treatment and comparator treatment employed as well as the dosing and regimens; primary endpoints. Measures of treatment effect were considered the proportion of participants obtaining a PASI 75 and a PASI 90. For dichotomous variables we expressed the results as risk ratios (RRs) and 95% confidence interval (CI). In addition, we calculated the number needed to treat (NNT) which represents the number of participants needed to be treated to achieve one additional positive outcome relative to the control group. We expressed the NNT for each treatment relative to placebo by the end of the primary assessment period. We summarized the included reports through descriptive analyses to provide an overview of studies’ characteristics, quality, effectiveness of the treatment investigated.

## Results

### Results of the Search

**FIGURE 1:**
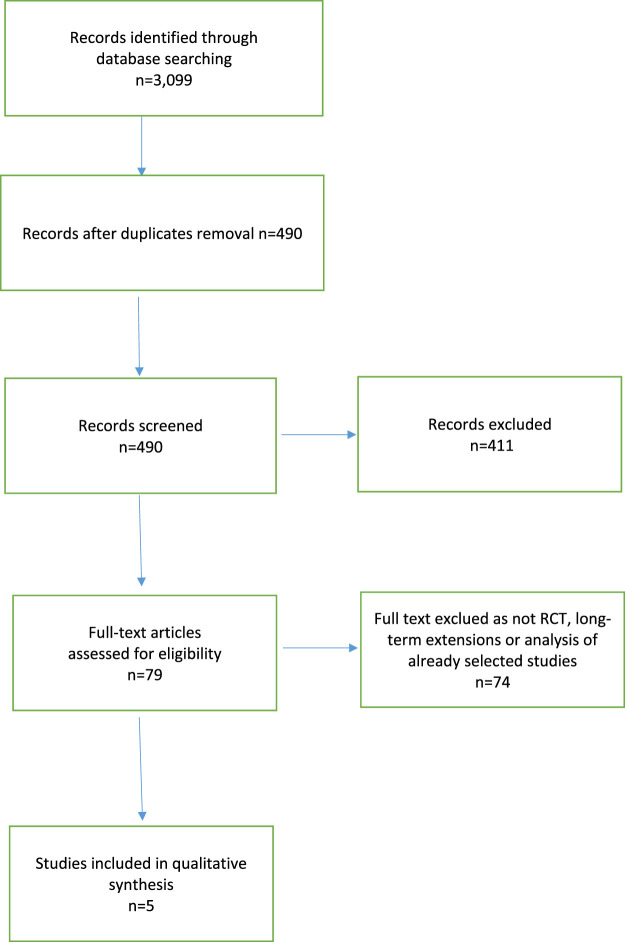
Study flow diagram.

We identified 3,099 articles matching the search criteria (Figure 1). After removing duplicates, 490 articles remained and were screened by title and abstract. Of the 79 articles that underwent full text screening, we retained five articles (Paller et al., 2008; Landells et al., 2015; Papp et al., 2017; Paller et al., 2020; Bodemer et al., 2021). Of these, all examined the efficacy of five biological drugs in the treatment of moderate-to severe psoriasis of children and/or adolescents. Basically, each of these five drugs has been evaluated in one study. The process of selecting articles for inclusion in this review is shown graphically in the flow diagram in Figure 1. The strengths of recommendations ratings, and the respective symbols used are summarised in Table 1.

**TABLE 1:** Strength of recommendation ratings, symbols used in subsequent statements and their corresponding definitions.

Strength of recommendation	Symbol	Definition
High	⊕⊕	High quality body of evidence from robust, large, well conducted trials, where benefits of treatment outweigh risks and adverse effects
Low	*	Low quality body of evidence from smaller studies, risks of bias where benefits of treatment, risks and adverse effects are closely matched
No Recommendation	X	Insufficient evidence
Against	-	Sufficient body of evidence where risks of treatment outweigh benefits

Baseline characteristics of the included studies are summarized in Table 2. Participants had stable moderate to severe plaque psoriasis at screening, defined as a PASI score equal to or greater than 12 in two studies (etanercept and ustekinumab trials), equal to or greater than 20 in the remaining three (adalimumab, secukinumab, ixekizumab trials); stable disease; PGA of at least three in two studies (etanercept and ustekinumab trials) or of at least four in the remaining three trials (adalimumab, secukinumab, ixekizumab trials); BSA involvement of at least 10% (etanercept, ustekinumab, secukinumab trials) or of at least 20% (adalimumab trial); a history of psoriasis in the last 6 months. The presence of psoriatic arthritis in enrolled patients was mentioned in three studies (Paller et al., 2008; Papp et al., 2017; Bodemer et al., 2021), but was not formally evaluated. A 75% improvement in the baseline PASI score, also known as a PASI 75 was the most frequently used primary endpoint in four out of five clinical trials (Paller et al., 2008; Papp et al., 2017; Paller et al., 2020; Bodemer et al., 2021). Only one study selected PGA 0/1 as primary endpoint and PASI 75 as secondary endpoint (Landells et al., 2015). PGA 0/1 (or alternatively IGA 0/1) was generally chosen as a co-primary endpoint to assess treatment efficacy (Papp et al., 2017; Paller et al., 2020; Bodemer et al., 2021). PASI 90 was a secondary endpoint in all five examined studies (Paller et al., 2008; Landells et al., 2015; Papp et al., 2017; Paller et al., 2020; Bodemer et al., 2021). PASI 100 was a secondary endpoint in three of out five studies (Papp et al., 2017; Paller et al., 2020; Bodemer et al., 2021). The primary end points were assessed at week 12 in four studies (Paller et al., 2008; Landells et al., 2015; Paller et al., 2020; Bodemer et al., 2021) and at 16 weeks in one study (Papp et al., 2017).

**TABLE 2:** Summary of main characteristics of the selected clinical trials.

Author/Study Acronim	Study design	Patient Eligibility	Dose	Number of Patients	Duration	Primary Endpoints	Statistical Analysis	Quality of Evidence (GRADE)
Paller (Paller et al., 2008)	Multicentre, double-blind	PASI≥12	⁃Etanercept 0.8 mg/kg (max 50 mg)	106	12 weeks	PASI 75	Intention to treat	⊕⊕ high
		PGA≥3	⁃Placebo	105				
		BSA≥10%						
Papp (Papp et al., 2017)	Multicentre, double-blind, multiperiod, phase 3 trial	PASI≥20 or ≥10 and at least one of active psoriatic arthritis unresponsive to non-steroidal anti-inflammatory drugs	⁃Adalimumab 0.8 mg/kg (max 40 mg)	38	16 weeks	PASI 75	Intention to treat	⊕⊕ high
		PGA≥4	⁃Adalimumab 0.4 mg/kg (max 20 mg)	39		PGA 0/1		
		BSA>20% or ≥10% with very thick lesions	⁃Methotrexate 0.1–0.4 mg/kg (max 25 mg per week total dose)	37				
		CDLQI≥10 or clinically relevant facial, genital, or hand or foot involvement						
Landells/CADMUS (Landells et al., 2015)	Multicentre, double-blind, phase 3 trial	PASI≥12	⁃Ustekinumab Standard Dose 0.75 mg/kg	36	12 weeks	PGA 0/1	Non responder imputation	⊕⊕ high
		PGA≥3	⁃Ustekinumab Half-Standard Dose 0.375 mg/kg	37				
		BSA≥10%	⁃Placebo	37				
Bodemer (Bodemer et al., 2021)	Multicentre, double-blind, placebo- and active controlled	PASI≥20	⁃Secukinumab low dose (LD) 150 mg	40	12 weeks	PASI 75	Non responder imputation	⊕⊕ high
		IGA≥4	⁃Secukinumab high dose (HD) 300 mg	40		IGA 0/1		
		BSA≥10	⁃Etanercept	41				
			⁃Placebo	41				
Paller/IXORA Ped (Paller et al., 2020)	Multicentre, double-blind, phase 3 trial	PASI ≥20 sPGA ≥4	⁃Ixekizumab^1^	115	12 weeks	PASI 75 sPGA 0/1	Non responder imputation	⊕⊕ high
			⁃Placebo	56				

According to body weight, dosing was as follows: subjects >50 kg received a starting dose of 160 mg, then 80 mg every 4 weeks (Q4W) thereafter; subjects 25–50 kg received a starting dose of 80 mg, then 40 mg Q4W thereafter; subjects <25 kg received a starting dose of 40 mg, then 20 mg Q4W thereafter).

### Anti-TNF-Alpha Agents

#### Etanercept (1 Trial)

In a placebo-controlled study on 211 patients, 4–17 years of age, Paller et al. (Paller et al., 2008) reported that etanercept 0.8 mg per kilogram of body weight (to a maximum of 50 mg) resulted in a greater percentage reduction in PASI 75 score versus placebo (57 vs. 11%, P=<0.001) at week 12. Similar results were observed for the secondary outcomes, with a higher proportion of reduction of PASI 50 (75 vs. 23%), PASI 90 (27 vs. 7%), and physician’s global assessment (PGA) of clear or almost clear (53 vs. 13%) in etanercept group vs. placebo (*p* < 0.001).

### Adalimumab (1 Trial)

Papp et al. (Papp et al., 2017) compared adalimumab 0.8 mg/kg or 0.4 mg/kg subcutaneously with oral methotrexate once weekly (0·1–0.4 mg/kg) for 16 weeks in 114 patients (aged ≥4 to <18 years). Adalimumab 0.8 mg/kg induced greater improvement in the PASI 75 score than methotrexate (58 vs. 32%, *p* = 0.027) and the clear or minimal PGA score (61 vs. 41%, *p* = 0.083) with respect to oral methotrexate. Adalimumab 0.8 mg/kg was also superior to oral methotrexate in the secondary efficacy end point of a PASI 90 response at week 16 (29 vs. 22%, *p* = 0.466), without statistical significance (Di Lernia, 2017; Papp et al., 2017).

### Anti-IL12/23 Agents

#### Ustekinumab (1 Trial)

Landells et al. (Landells et al., 2015) compared in the CADMUS trial ustekinumab standard dosing (0.75 mg/kg for patients ≤60 kg, 45 mg for patients 60-≤100 kg, and 90 mg for patients >100 kg) or half-standard dosing (0.375 mg/kg for patients ≤60 kg, 22.5 mg for patients 60-100 kg, and 45 mg for patients >100 kg) versus placebo during 12 weeks of treatment in 110 patients aged 12–17 years. Treatment with ustekinumab standard and half standard dosing respectively resulted in significantly better percentage improvement in the primary endpoint PGA score 0/1 than the placebo group (69.4 and 67.6% vs. 5.4%, *p* < 0.001). Similarly, using ustekinumab standard and half standard dosing respectively resulted in significant improvement also for major secondary endpoints compared with placebo, in particular for PASI 75 (80.6 and 78.4% vs. 10.8%, *p* < 0.001), PASI 90 (61.1 and 54.1% vs. 5.4%, *p* < 0.001) and CDLQI (-6.7 and -5.6 vs. -1.5, *p* < 0.01).

### Anti-IL17 Agents

#### Secukinumab (1 Trial)

Bodemer et al. (Bodemer et al., 2021) compared secukinumab (low dose, 150 mg, and high dose, 300 mg) with placebo and etanercept in 162 patients aged six to <18 years during 12 weeks. The co-primary objectives of the study were met with both secukinumab doses. Treatment with low and high dose secukinumab respectively compared with placebo resulted in greater improvement in the PASI 75 score (80% and 77,5 vs. 14.6%, *p* < 0.0001), IGA 0/1 (70 and 60% vs. 4.9%, *p* < 0.0001). In addition, both secukinumab dose groups (low and high dose) respectively achieved significantly higher (*p* < 0.05) response versus etanercept with respect to IGA 0/1 (70.0 and 60% versus 34.1%) and PASI 90 (72.5 and 67.5% versus 29.3%). Treatment with low and high dose secukinumab compared with placebo resulted in significant improvements in other secondary endpoints as well, as PASI 100 (30.0 and 27.5% vs. 0%) and CDLQI 0/1 (44.7 and 50% vs. 15%, P 0.05 and 0.001).

### Ixekizumab (1 Trial)

In a placebo-controlled study (IXORA-PEDs) with 171 patients aged six to <18 years, Paller et al. (Paller et al., 2020) highlighted that ixekizumab resulted in significantly better percentage improvement in the primary endpoints PASI 75 and sPGA 0/1 respectively than the placebo group (PASI 75 89% vs. placebo 25%, *p* < 0.0001) (sPGA 81 versus 11%). Ixekizumab was also superior for all secondary endpoints, including PASI 90 (78% versus placebo 5%) PASI 75 and sPGA (0,1) at week 4, improvement in itch, and complete skin clearance.

Efficacy outcomes relative to PASI 75 and PASI 90 are presented respectively in Table 3; Table 4. A two-sided asymptotic 95% CI was calculated for the relative risk (RR) of a PASI 75 and PASI 90 response at week 12 or at week 16 for adalimumab/methotrexate. RR of PASI 75 response was 7.45 for ustekinumab, 5.47 for secukinumab, 4.95 for etanercept, 3.55 for ixekizumab, 1.79 for adalimumab (Table 3). RR of PASI 90 response was 29.73 for secukinumab, 11.22 for ixekizumab, 9.47 for ustekinumab, 3.28 for etanercept, 1.34 for adalimumab (Table 4).

**TABLE 3:** Efficacy outcome: PASI 75. Active treatment (intervention) compared to placebo or active comparator (methotrexate, etanercept).

Publication	Intervention	Illustrative Comparative Risks*		Relative risk (95% CI)	No of Participants**	Number needed to treat (NNT)
		Assumed risk	Corresponding risk			
Paller (Paller et al., 2008)	Etanercept	**Placebo**	**Etanercept**	RR 4.95 (2.83–8.65)	211	2.2
		114 per1000	566 per1000			
Papp (Papp et al., 2017)	Adalimumab	**Methotrexate**	**Adalimumab 0.8 mg/kg**	RR 1.79 (1.04–3.06)	75	3.8
		324 per1000	578 per1000			
Landells (Landells et al., 2015)	Ustekinumab	**Placebo**	**Ustekinumab standard dose**	RR 7.45 (2.91–19.06)	73	1.4
		108 per1000	805 per1000			
Bodemer (Bodemer et al., 2021)	Secukinumab	**Placebo**	**Secukinumab Low dose**	RR 5.47 (2.57–11.64)	81	1.5
		146 per1000	800 per1000			
		**Etanercept**	**Secukinumab Low dose**	RR 3.17 (2.20–4.57)	81	6
		634 per1000	800 per1000			
Paller (Paller et al., 2020)	Ixekizumab	**Placebo**	**Ixekizumab**	RR 3.55 (2.24–5.61)	171	1.6
		250 per1000	887 per1000			

*Illustrative comparative risk is presented in the form of a number of people experiencing the chosen event (PASI, 75) per 1,000 persons. The assumed risk is calculated in a group of people (placebo or active comparator group) who did not receive the intervention (drug under investigation). The corresponding risk is calculated in the group who received the intervention.

**Number of participants involved in the efficacy outcome may differ from total number of trial participants due to the presence of more than two groups of patients treated with different drug dosages in some of the selected studies (24–25).

**TABLE 4:** Efficacy outcome: PASI 90. Active treatment (intervention) compared to placebo or active comparator (methotrexate, etanercept).

Publication	Intervention	Illustrative Comparative Risks*		Relative Risk (95% CI)	No of Participants **	Number Needed to Treat (NNT)
		Assumed risk	Corresponding risk			
Paller (Paller et al., 2008)	Etanercept	**Placebo**	**Etanercept**	RR 3.28 (1.51–7.12)	211	5
		67 per1000	273 per1000			
Papp (Papp et al., 2017)	Adalimumab	**Methotrexate**	**Adalimumab 0.8 mg/kg**	RR 1.34 (0.61–2.95)	75	14.3
		216 per1000	289 per1000			
Landells (Landells et al., 2015)	Ustekinumab	**Placebo**	**Ustekinumab standard dose**	RR 9.47 (2.42–37.11)	73	1.8
		54 per1000	611 per1000			
Bodemer (Bodemer et al., 2021)	Secukinumab	**Placebo**	**Secukinumab low dose**	RR 29.73 (4.25–207.90)	81	1.4
		24 per1000	725 per1000			
		**Etanercept**	**Secukinumab low dose**	RR 2.48 (1.48–4.14)	81	2.3
		293 per1000	725 per1000			
Paller (Paller et al., 2020)	Ixekizumab	**Placebo**	**Ixekizumab**	RR 11.22 (3.75–33.55)	171	1.4
		53 per1000	782 per1000			

* Illustrative comparative risk is presented in the form of a number of people experiencing the chosen event (PASI, 90) per 1,000 persons. The assumed risk is calculated in a group of people (placebo o active comparator group) who did not receive the intervention (drug under investigation). The corresponding risk is calculated in the group who received the intervention.

**Number of participants involved in the efficacy outcome may differ from total number of trial participants due to the presence of more than two groups of patients treated with different drug dosages in some of the selected studies (Landells et al., 2015; Papp et al., 2017).

In addition, the NNT for the outcome PASI 75 and PASI 90 in the exposed groups compared to those receiving placebo (Paller et al., 2008; Landells et al., 2015; Paller et al., 2020; Bodemer et al., 2021) or methotrexate and etanercept (Landells et al., 2015; Papp et al., 2017) have been exploited. NNT for PASI 75 response was 1.4 for ustekinumab, 1.5 for secukinumab, 1.6 for ixexizumab, 2.2 for etanercept, 3.8 for adalimumab (Table 3). NNT for PASI 90 response was 1.4 for secukinumab and ixekizumab, 1.8 for ustekinumab, five for etanercept, 14.3 for adalimumab (Table 4)

## Discussion

The exact role of biologics in the treatment of paediatric psoriasis is evolving (Napolitano et al., 2016). This systematic review summarizes the up-to-date evidence on the short-term efficacy of licensed biologic therapies in the treatment of paediatric psoriasis.

Five studies referring to five biologic agents for moderate-to-severe psoriasis were included.

We selected PASI 75 as our primary outcome due its relatively wide use in current psoriasis trials, and PASI 90. The measure of improvement in a patient-oriented score, such as CDLQI, has not been investigated.

Heterogeneity of patient age and psoriasis severity across the selected five studies was found. Younger enrolled patients were children aged four in one study (Papp et al., 2017), six in three studies (Paller et al., 2008; Paller et al., 2020; Bodemer et al., 2021) and 12 in one study (Landells et al., 2015). PASI baseline was ≥20 in three studies (Papp et al., 2017; Paller et al., 2020; Bodemer et al., 2021) and ≥12 in the remaining two (Paller et al., 2008; Landells et al., 2015).

Effect size estimates suggested that etanercept, ustekinumab, secukinumab and ixekizumab reduced overall psoriasis symptoms more than placebo; secukinumab more than etanercept; adalimumab more than methotrexate. The latter result was not statistically significant probably due to the limited sample size and power of the study (Di Lernia, 2017).

According to RR, when compared with placebo the decreasing rank order for PASI 75 endpoint was ustekinumab, secukinumab, etanercept, ixekizumab, for PASI 90 endpoint secukinumab, ixekizumab, ustekinumab, etanercept. Noteworthy, PASI 75 response rate of ixekizumab was higher than etanercept, but the high placebo PASI 75 response rate (Paller et al., 2020) had influence on the assessment.

NNT can help to quantify efficacy outcomes and give support to place various therapeutic options into clinical perspective. NNT for additional benefit on the PASI 75 outcome showed as the most effective options ustekinumab, secukinumab and ixekizumab with very low difference among them. NNT for additional benefit on PASI 90 showed as the most effective option secukinumab and ixekizumab.

## Conclusion

In this study, we highlighted that the available biologic therapies for psoriasis are efficacious for paediatric psoriasis. Our study showed that there was a trend to better response to certain biological classes and in particular that anti-IL-17 agents seem to be superior to anti-TNF-alpha therapies in the treatment of paediatric psoriasis, consistent with their corresponding efficacy in adults (Fahrbach et al., 2021; Sbidian et al., 2021). However, all the drugs were compared in a blinded, randomised comparison in the short term.

Finally, long term observations and large registries are necessary to enhance our knowledge about efficacy and safety of these drugs.

## Data Availability

The original contributions presented in the study are included in the article/Supplementary Material, further inquiries can be directed to the corresponding author.

## References

[B1] Augustin M. Glaeske G. Radtke M. A. Christophers E. Reich K. Schäfer I. (2010). Epidemiology and Comorbidity of Psoriasis in Children. Br. J. Dermatol. 162, 633–636. 10.1111/j.1365-2133.2009.09593.x 19922529

[B2] Bodemer C. Kaszuba A. Kingo K. Tsianakas A. Morita A. Rivas E. (2021). Secukinumab Demonstrates High Efficacy and a Favourable Safety Profile in Paediatric Patients with Severe Chronic Plaque Psoriasis: 52-week Results from a Phase 3 Double-Blind Randomized, Controlled Trial. J. Eur. Acad. Dermatol. Venereol. 35, 938–947. 10.1111/jdv.17002 33068444 PMC7986088

[B3] Cannavò S. P. Guarneri F. Giuffrida R. Aragona E. Guarneri C. (2017). Evaluation of Cutaneous Surface Parameters in Psoriatic Patients. Skin Res. Technol. 23 (1), 41–47. 10.1111/srt.12299 27270565

[B4] Charbit L. Mahé E. Phan A. Chiaverini C. Boralevi F. Bourrat E. (2016). Systemic Treatments in Childhood Psoriasis: a French Multicentre Study on 154 Children. Br. J. Dermatol. 174, 1118–1121. 10.1111/bjd.14326 26617180

[B5] Cvenkel K. Starbek Zorko M. (2021). Challenges in the Treatment of Psoriasis in Childhood. Acta Dermatovenerol Alp Pannonica Adriat 30, 105–108. 10.15570/actaapa.2021.26 34565125

[B6] Di Lernia V. (2017). Adalimumab for Treating Childhood Plaque Psoriasis: a Clinical Trial Evaluation. Expert Opin. Biol. Ther. 17, 1553–1556. 10.1080/14712598.2017.1369950 28829204

[B7] Di Lernia V. Bonamonte D. Lasagni C. Belloni Fortina A. Cambiaghi S. Corazza M. (2016). Effectiveness and Safety of Acitretin in Children with Plaque Psoriasis: A Multicenter Retrospective Analysis. Pediatr. Dermatol. 33, 530–535. 10.1111/pde.12940 27443789

[B8] Di Lernia V. Neri I. Calzavara Pinton P. Di Nuzzo S. Stingeni L. Guarneri C. (2017). Treatment Patterns with Systemic Antipsoriatic Agents in Childhood Psoriasis: an Italian Database Analysis. G Ital. Dermatol. Venereol. 152, 327–332. 10.23736/S0392-0488.16.05287-X 26761766

[B9] Di Lernia V. Stingeni L. Boccaletti V. Calzavara Pinton P. G. Guarneri C. Belloni Fortina A. (2016). Effectiveness and Safety of Cyclosporine in Pediatric Plaque Psoriasis: A Multicentric Retrospective Analysis. J. Dermatolog Treat. 27, 395–398. 10.3109/09546634.2015.1120852 26571044

[B10] Fahrbach K. Sarri G. Phillippo D. M. Neupane B. Martel S. E. Kiri S. (2021). Short-Term Efficacy of Biologic Therapies in Moderate-To-Severe Plaque Psoriasis: A Systematic Literature Review and an Enhanced Multinomial Network Meta-Analysis. Dermatol. Ther. (Heidelb) 11, 1965–1998. 10.1007/s13555-021-00602-z 34549383 PMC8611163

[B11] Finlay A. Y. (2005). Current Severe Psoriasis and the Rule of Tens. Br. J. Dermatol. 152, 861–867. 10.1111/j.1365-2133.2005.06502.x 15888138

[B12] Fortina A. B. Bardazzi F. Berti S. Carnevale C. Di Lernia V. El Hachem M. (2017). Treatment of Severe Psoriasis in Children: Recommendations of an Italian Expert Group. Eur. J. Pediatr. 176, 1339–1354. 10.1007/s00431-017-2985-x 28836064

[B13] Gisondi P. Talamonti M. Chiricozzi A. Piaserico S. Amerio P. Balato A. (2021). Treat-to-Target Approach for the Management of Patients with Moderate-To-Severe Plaque Psoriasis: Consensus Recommendations. Dermatol. Ther. (Heidelb) 11 (1), 235–252. 10.1007/s13555-020-00475-8 33426634 PMC7859133

[B14] Ingrasciotta Y. Isgrò V. Ientile V. Tari M. Trifirò G. Guarneri C. (2021). Are Patients with Psoriasis and Psoriatic Arthritis Undertreated? A Population-Based Study from Southern Italy. J. Clin. Med. 10 (15), 3431. 10.3390/jcm10153431 34362214 PMC8348176

[B15] Kim E. Fischer G. (2021). Relationship between PASI and FDLQI in Paediatric Psoriasis, and Treatments Used in Daily Clinical Practice. Australas. J. Dermatol. 62, 190–194. 10.1111/ajd.13536 33586132

[B16] Landells I. Marano C. Hsu M. C. Li S. Zhu Y. Eichenfield L. F. (2015). Ustekinumab in Adolescent Patients Age 12 to 17 Years with Moderate-To-Severe Plaque Psoriasis: Results of the Randomized Phase 3 CADMUS Study. J. Am. Acad. Dermatol. 73, 594–603. 10.1016/j.jaad.2015.07.002 26259989

[B17] Lavaud J. Mahé E. (2019). Scores de sévérité dans le psoriasis de l'enfant : revue systématique de la littérature. Ann. de Dermatologie de Vénéréologie 146, 771–782. 10.1016/j.annder.2019.03.007 31060749

[B18] Marcianò I. Randazzo M. P. Panagia P. Intelisano R. Sgroi C. Ientile V. (2020). Real-world Use of Biological Drugs in Patients with Psoriasis/psoriatic Arthritis: a Retrospective, Population-Based Study of Years 2010-2014 from Southern Italy. G Ital. Dermatol. Venereol. 155 (4), 441–451. 10.23736/S0392-0488.18.05753-X 29582617

[B19] Moher D. Shamseer L. Clarke M. Ghersi D. Liberati A. Petticrew M. (2015). Preferred Reporting Items for Systematic Review and Meta-Analysis Protocols (PRISMA-P) 2015 Statement. Syst. Rev. 4, 1. 10.1186/2046-4053-4-1 25554246 PMC4320440

[B20] Napolitano M. Megna M. Balato A. Ayala F. Lembo S. Villani A. (2016). Systemic Treatment of Pediatric Psoriasis: A Review. Dermatol. Ther. (Heidelb) 6, 125–142. 10.1007/s13555-016-0117-6 27085539 PMC4906111

[B21] Paller A. S. Seyger M. M. B. Alejandro Magariños G. Bagel J. Pinter A. Cather J. (2020). Efficacy and Safety of Ixekizumab in a Phase III, Randomized, Double-Blind, Placebo-Controlled Study in Paediatric Patients with Moderate-To-Severe Plaque Psoriasis (IXORA-PEDS). Br. J. Dermatol. 183, 231–241. 10.1111/bjd.19147 32316070 PMC7496501

[B22] Paller A. S. Siegfried E. C. Langley R. G. Gottlieb A. B. Pariser D. Landells I. (2008). Etanercept Treatment for Children and Adolescents with Plaque Psoriasis. N. Engl. J. Med. 358, 241–251. 10.1056/NEJMoa066886 18199863

[B23] Papp K. Thaçi D. Marcoux D. Weibel L. Philipp S. Ghislain P. D. (2017). Efficacy and Safety of Adalimumab Every Other Week versus Methotrexate once Weekly in Children and Adolescents with Severe Chronic Plaque Psoriasis: a Randomised, Double-Blind, Phase 3 Trial. Lancet 390, 40–49. 10.1016/S0140-6736(17)31189-3 28478975

[B24] Parisi R. Symmons D. P. Griffiths C. E. Ashcroft D. M. (2013). Global Epidemiology of Psoriasis: a Systematic Review of Incidence and Prevalence. J. Invest. Dermatol. 133, 377–385. 10.1038/jid.2012.339 23014338

[B25] Philipp S. Menter A. Nikkels A. F. Barber K. Landells I. Eichenfield L. F. (2020). Ustekinumab for the Treatment of Moderate-To-Severe Plaque Psoriasis in Paediatric Patients (≥ 6 to. Br. J. Dermatol. 183, 664–672. 10.1111/bjd.19018 32173852

[B26] Raychaudhuri S. P. Gross J. (2000). A Comparative Study of Pediatric Onset Psoriasis with Adult Onset Psoriasis. Pediatr. Dermatol. 17, 174–178. 10.1046/j.1525-1470.2000.01746.x 10886746

[B27] Sbidian E. Chaimani A. Afach S. Doney L. Dressler C. Hua C. (2021). Systemic Pharmacological Treatments for Chronic Plaque Psoriasis: a Network Meta-Analysis. Cochrane Database Syst. Rev. 1 (4), CD011535. 10.1002/14651858.CD011535.pub3 PMC8408312 33871055

[B28] Spuls P. I. Lecluse L. L. Poulsen M. L. Bos J. D. Stern R. S. Nijsten T. (2010). How Good Are Clinical Severity and Outcome Measures for Psoriasis?: Quantitative Evaluation in a Systematic Review. J. Invest. Dermatol. 130, 933–943. 10.1038/jid.2009.391 20043014

[B29] Talamonti M. Galluzzo M. Chiricozzi A. Quaglino P. Fabbrocini G. Gisondi P. (2020). Management of Biological Therapies for Chronic Plaque Psoriasis during COVID-19 Emergency in Italy. J. Eur. Acad. Dermatol. Venereol. 34 (12), e770–e772. 10.1111/jdv.16841 32735716 PMC7436412

[B30] Tollefson M. M. Crowson C. S. McEvoy M. T. Maradit Kremers H. (2010). Incidence of Psoriasis in Children: a Population-Based Study. J. Am. Acad. Dermatol. 62, 979–987. 10.1016/j.jaad.2009.07.029 19962785 PMC3818908

[B31] van Geel M. J. Oostveen A. M. Hoppenreijs E. P. Hendriks J. C. van de Kerkhof P. C. de Jong E. M. (2015). Methotrexate in Pediatric Plaque-type Psoriasis: Long-Term Daily Clinical Practice Results from the Child-CAPTURE Registry. J. Dermatolog Treat. 26, 406–412. 10.3109/09546634.2014.996515 25485870

